# A Case of Lithotomy

**Published:** 1879-06

**Authors:** John Godfrey

**Affiliations:** Assistant Surgeon Marine Hospital Service, Mobile, Ala.


### A CASE OF LITHOTOMY.

By JOHN GODFREY, M.D.,
Assistant Surgeon Marine Hospital Service, Mobile, Ala.

The following case is reported principally as a contribution to the statistics of operations of this class, and as an additional argument to cut without delay whenever the accident which necessitated the operation should occur, forasmuch as such accidents have probably not happened for the last time. For, notwithstanding the patient was finally brought out of his difficulty, an immediate extraction in the way it was done would have saved him many months of extreme anxiety and suffering:

On the 25th of September, 1878, I took charge of the Marine Hospital in Mobile, and found among those on the sick list seaman Thomas, suffering from cystic irritation. He had been cut for stricture with the Maisonneuve instrument about six months previously, at which time, unfortunately, the filiform was incised and a part of it left in the bladder.

Repeated attempts were made immediately after and at various subsequent intervals to remove the body, but without avail. Irritation of the bladder soon set in, giving rise to the usual symptoms of vesical calculus. Not long before I saw the patient he had begun to pass small calculous concretions, about half the size of a rice grain, and soon thereafter a number of pieces with a hole in the centre, showing that they had been moulded about the foreign body and had become detached.

At this time micturition was constant, difficult and attended by a great deal of pain, and altogether his condition was quite distressing.

Sound exploration revealed calculous formation, but following the example of my predecessor, I also made an effort to extract *per urethram;* as was expected, failure ensued. I now determined to perform lithotomy. Meantime yellow fever began to spread; and partly on account of the engrossing nature of that disease, and the suspicion that such surgery as could wait should be deferred until its subsidence, and particularly on account of the debilitated and irritable condition of the patient, the operation was postponed till the 25th of November, at which time he was thought to be built up sufficiently to stand it.

With the assistance of Drs. Goode and Fournier, Surgeon Sutton, Marine Hospital Service, giving the chloroform, and Dr. Scales controlling the staff, I opened the bladder by the lateral method and succeeded in bringing away after many tedious attempts, the filiform, to which were attached thirteen calculi—or rather twelve—one coming off in the wound and removed by the hand.

As intimated, the operation lasted longer than usual; owing in part to the peculiar shape of the body, within the bladder, which I found unexpectedly to be curled upon itself, and slightly agglutinated by a species of false membrane, quite thin and very slippery; in part to ill luck or lack of cunning in getting the ever-shifting substance within the blades of the forceps. The bladder was then carefully and effectually syringed by my friend Dr. Goode, the patient put to bed, and a skilled attendant set to watch him.

The further history of the case contains little of interest. For the first half day there was a good deal of nausea. Three hours after the operation two slight rigors came on lasting only a few minutes each, followed by inconsiderable rise of temperature. The second day the patient had diarrhœa which yielded to treatment the day following. Up to the fifth day the temperature ranged from three-fourths to one and a half degrees above normal; pulse from eighty to ninety-five. From that time everything went smoothly. The wound healed kindly, and in three weeks the patient was going about the ward.

The concretions are remarkably light, and appear to be of the ammonia-magnesia-phosphate variety. * * * * —*N. O. Med. and Surg. Journal.*

